# Supplementary material for: Where did the river go? Testing the hypothesis of rivers discharging into the Gulf of Sirt (East Mediterranean) during the late Pleistocene
Source: PLoS One. 2025 Oct 8;20(10):e0331681. doi: 10.1371/journal.pone.0331681 (PMC12507219; doi:10.1371/journal.pone.0331681)
Supplement: S1 File — (PDF) [file pone.0331681.s001.pdf]

Supporting Information

Where did the river go? Testing the hypothesis of rivers discharging into the Gulf of Sirt (East Mediterranean) during the late Pleistocene

B Mauz, E Abdulsamad, S Emhanna, N Elmejdoub, M Mansoura, M Rogerson

1 Field work

No specific permits were required to conduct the fieldwork, as the study area consists of open public land that is neither protected nor classified as a nature reserve or restricted zone. These locations are freely accessible to the general public and are commonly used for educational and scientific purposes. Sampling in such areas does not typically require formal authorisation from governmental or environmental agencies.

2 Stratigraphy and spatial occurrence of Plio-Pleistocene sediments

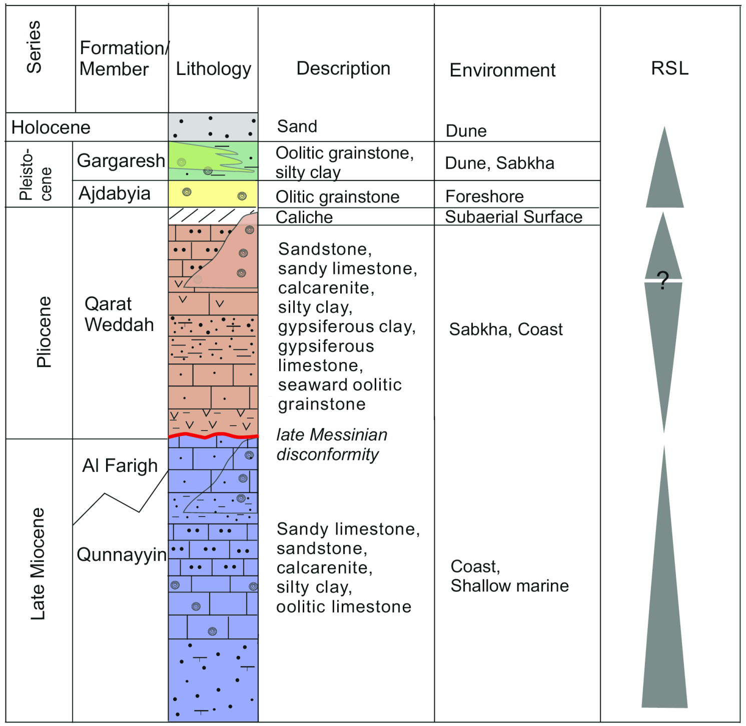

Fig S1. The stratigraphy of the Ajdabiya trough (modified after [37-38]); RSL = relative sea level.

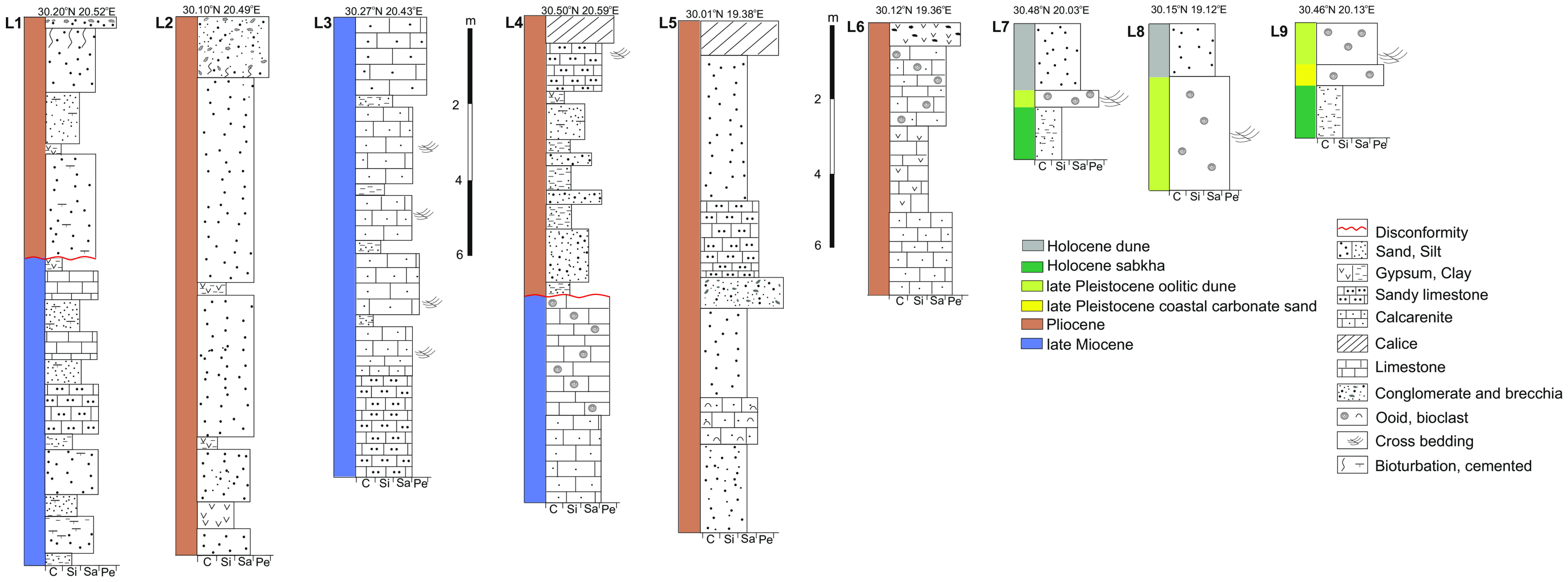

Fig S2. Logs of the Ajdabiya Trough (redrawn from [38]). For location of logs see Fig S3.

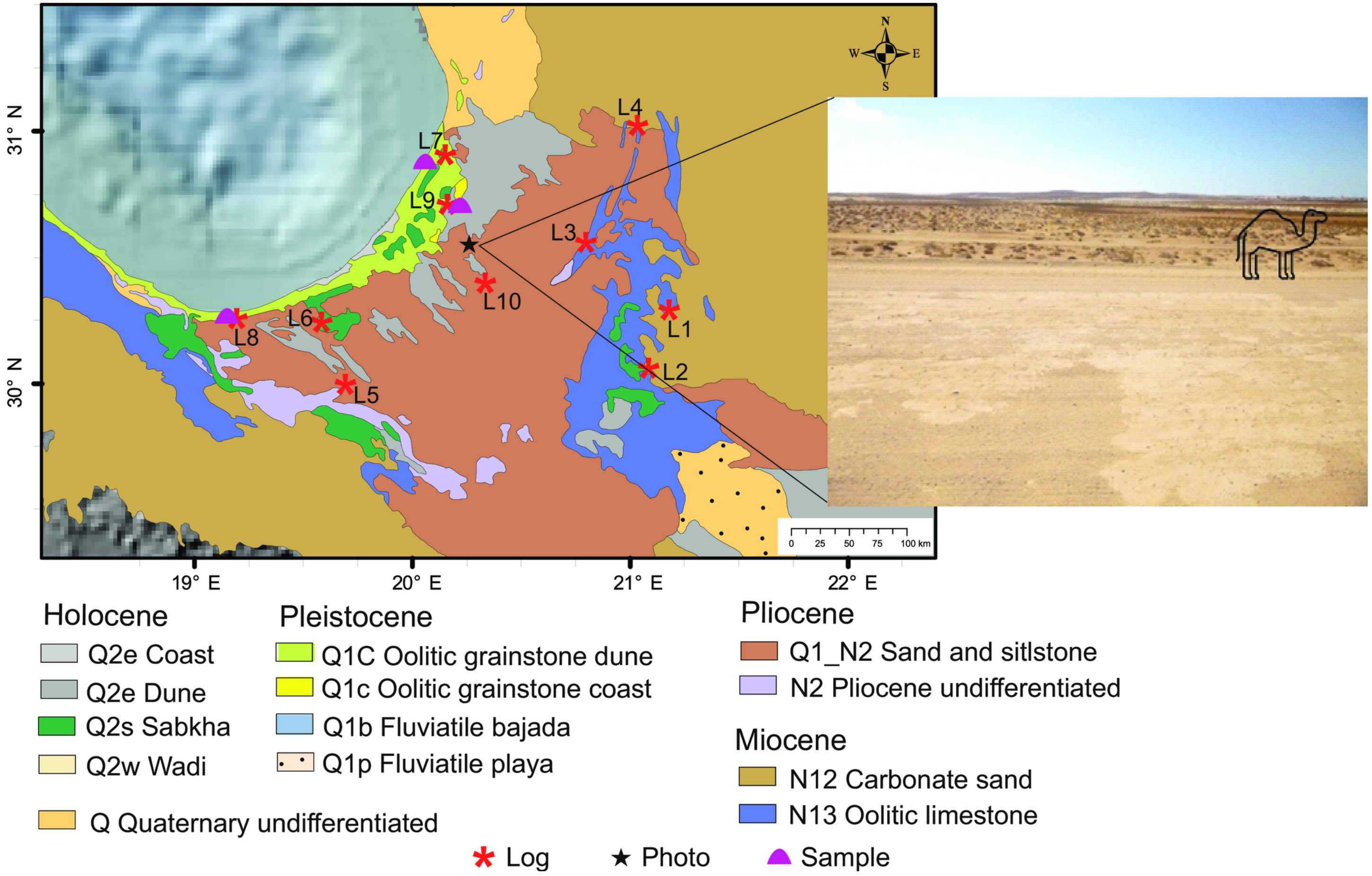

Fig S3. Geological map of the Ajdabiya Trough with location of logs shown in Fig S2. Photo shows the NE landscape looking NW with the coastal dune belt in the background. Samples are those listed in Tables S1 and S2. Background data of the map reprinted from NOAA National Centers for Environmental Information 2022: ETOPO 2022 15 Arc-Second Global Relief Model. DOI: 10.25921/fd45-gt74 under a CC BY licence.

3 Ooids

Ooids are (sub)spherical carbonate grains (see Fig S5) which form through inorganic precipitation of aragonite around a nucleus (e.g., quartz grain). The inorganic precipitation requires seawater that is supersaturated with respect to $CaCO_2$, so the inhibiting effect of $MgCO_3$ ion-pair is overcome. The process typically occurs in alkaline, shallow and warm water to allow for microbial activity (Morse and Mackenzie, 1990). The cortex of the Sirt ooids show tangential orientation of aragonite needles which may result from the adsorption of colloidal-sized aragonite on the surface of the nucleus with their longest axis parallel to the surface. This, being the most stable arrangement of the cortex (Bathurst, 1967), requires rolling of the grain on the seabed, hence agitated water where local water agitation equals the maximum non-coated grain. Smaller grains are coated until they reach the maximum grain size sustained by the local water energy.

The carbonate production is sensitive to sea-surface temperature and salinity. It would have ceased under large-scale freshwater input into the coastal water due to drop of salinity and alkalinity. For more information on ooids see review of Simone (1980) and Trower (2020) and Koeshidayatullah et al. (2022) for climate significance.

Modern analogues for ooids are the Arabian Gulf, the Gulf of Aqaba, the Gulf of Suez, the Bahama Bank and adjacent Caicos Bank (for details about these analogues see [52]). In the Mediterranean Pleistocene and mid-Holocene oolitic sediments occur on its west, south and east coasts [52]. No ooids form on modern Mediterranean coasts.

Table S1. Description of coastal sediments derived from thin section analysis. In all samples cortex laminae orientation (i.e. the c-axis of aragonitic laminae with respect to the nucleus surface) of ooids is tangential. Ooid nuclei are quartz and bioclasts.

**Sample code**	**Ooids**			**Dominant components**	**Matrix**	**Sediment classification**	**Texture**	**Environment of deposition**	**Site reference (lat/long)**
	Ooid size (μm)	Cortex thickness (μm)	Cortex mineral						
B3	200	63	Ar	peloids, ooids	Some bladed cr	oolitic grainstone	well sorted, subrounded	backshore	30°48'/20°06'
B9	490	300	Ar	ooids	Fibrous isopachous rim	oolitic grainstone	mod to poorly sorted, subrounded	foreshore	30°40'/20°10'
LV708	170	40	?	peloids, ooids	Isopachous rim, bladed crystals	oolitic grainstone	mod to poorly sorted, rounded	backshore	30°15'/19°11'

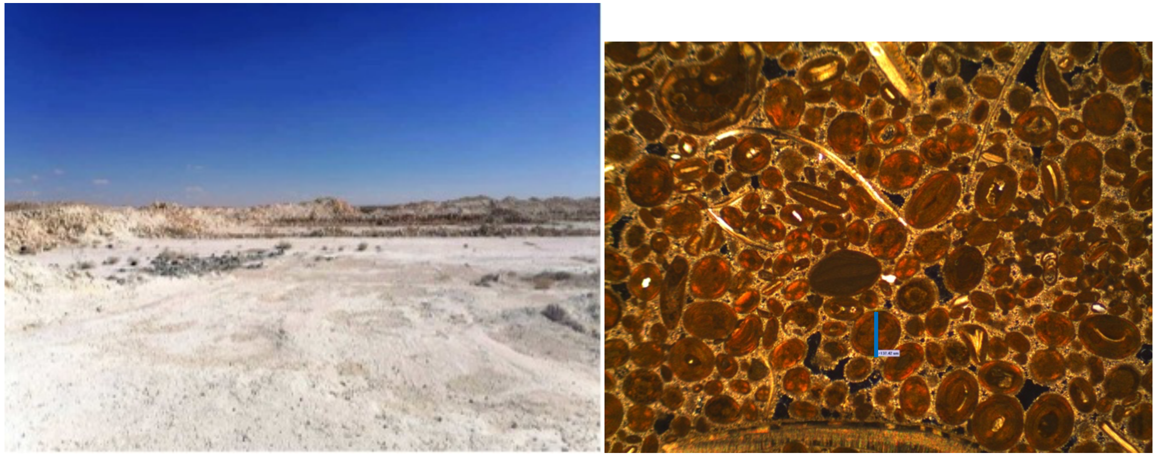

Fig S4. Left: The quarry close to Ajdabiya town showing the oolitic grainstone deposits (Ajdabiya Fm) assigned to MIS 5e [38]; Right: thin section of the oolitic grainstone (sample B9 in Table S1; blue bar is 137 µm long) showing concentric aragonitic rings around a nucleus. Fibrous isopacheous rim cement indicate early diagenesis.

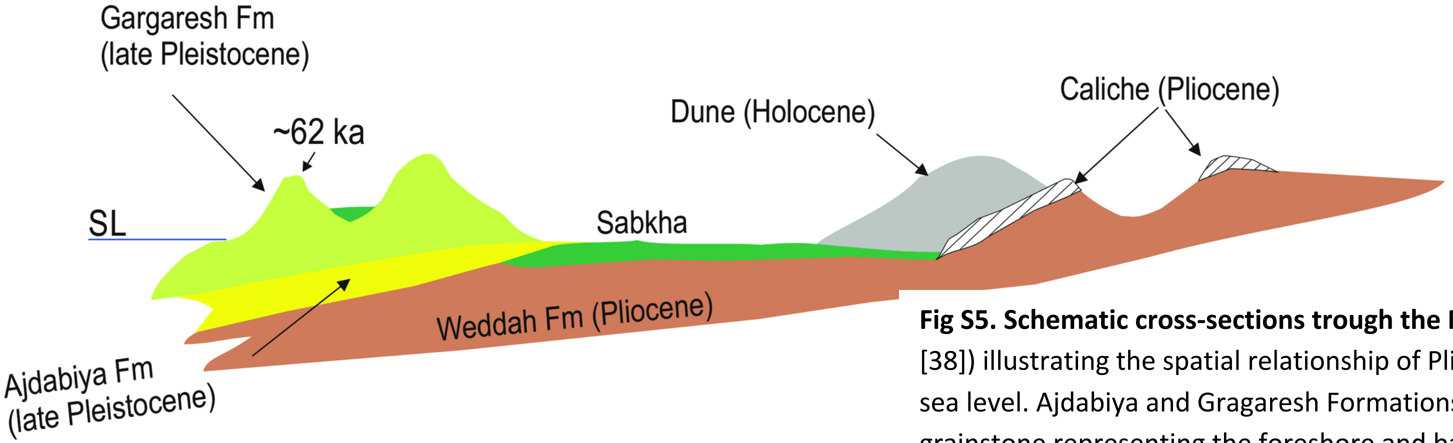

Fig S5. Schematic cross-sections trough the NE Ajdabiya trough (modified from [38]) illustrating the spatial relationship of Plio-Pleistocene strata; SL=modern sea level. Ajdabiya and Gragaresh Formations (Fm) are composed of oolitic grainstone representing the foreshore and backshore environment, respectively.

4 The Ajdabiya-Tobrouq Ridge

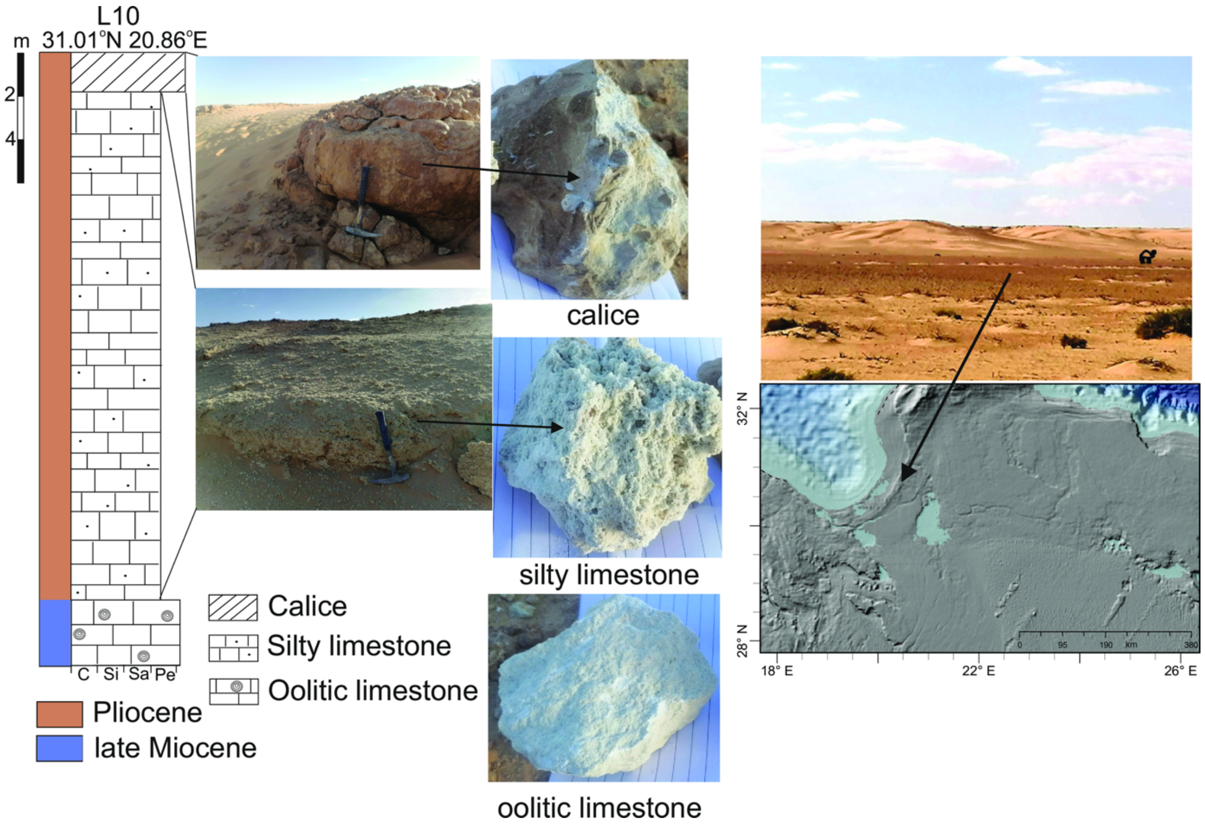

Fig S6. Description of the Ajdabiya-Tobrouq Ridge showing the log and lithological data and the landscape of the ridge looking northwest. For location of log L 10 see Fig S3. Digital elevation model reprinted from NOAA National Centers for Environmental Information, 2022: ETOPO 2022 15 Arc-Second Global Relief Model. DOI: 10.25921/fd45-gt74 under a CC BY licence.

5 Optical dating

Optically stimulated luminescence dating relies on the availability of 90-300 mm quartz grains younger than their respective age limit which is typically around 150 ka. The four steps required to date a quartz sample comprise: (1) preparing sample in the laboratory under subdued light in order to extract quartz grains of

suitable size, (2) testing the quartz sub-sample to find the suitable measurement protocol and deploying this protocol to *n* aliquots (see Table 1) until data show consistent skewness, (3) analysing the data using standard rejection criteria of aliquot data and assess the dose distribution, (4) determining the annual dose rate using low-level gamma spectrometry and relevant conversion factors and calculate the age (Table S2). The total uncertainty of the age is the sum of experimental errors and systematic uncertainties and expressed as 1σ standard error. For details of technical approach see Mauz et al. (2002), Mauz et al. (2022), Murray et al. (2021).

Table S2. Analytical data used to determine optical ages. OD stands for over-dispersion, i.e. the percentage of data that lies outside the 2σ standardised estimate. OD is typically >10% due to intrinsic variability of quartz properties relevant for dating.

**Sample code**	**Sample Field**	**Location**	***n* Aliquot (accepted/ measured)**	**Grain Size (µm)**	**Aliquot Size (mm)**	**OD (%)**	**D_e (median) ± 1σ (Gy)**	**OSL Age (±1σ, ka)**
LV 708	C1-S3	30°15’ N 19°11’ E	55/72	200-250	3	16 ± 3	55.6 ± 1.3	61.5 ± 1.6
LV 754	Chott	33°46’ N 9°05’ E	40/96	150-200	3	29 ± 4	160 ± 8	199 ± 10

Table S3. Radioisotope concentration and potassium weight percent determined in the samples. The total dose rate ($\dot{D}$) was calculated using conversion factors of Guérin et al., 2011 and attenuation factors of Guérin and Mercier 2012. Both samples were collected at modern ground surface resulting in 22-26% of the total dose rate being due to the cosmic dose rate ($\dot{D}_{cosm}$).

**Sample Code**	**U (ppm)**	**Th (ppm)**	**K (wt %)**	**$\dot{D}_{cosm}$ (Gy ka^{-1})**	**$\dot{D}$ (Gy ka^{-1})**
LV 708	2.00 ± 0.05	1.09 ± 0.09	0.140 ± 0.009	0.20 ± 0.01	0.91 ± 0.001
LV 754	0.69 ± 0.02	0.64 ± 0.08	0.16 ± 0.01	0.21 ± 0.01	0.80 ± 0.02

References

Morse JW and Mackenzie FT. Geochemistry of sedimentary carbonates. Developments in Sedimentology 1990, 48, Elsevier Amsterdam, New York

Bathurst RGC. Oolitic films on low energy carbonate sand grains, Bimini Lagoon, Bahamas. Marine Geology 1967, 5, 89-109.

Simone L. Ooids: a review. Earth-Science Reviews 1980, 16, 319-355.
Trower, E. J.. (2020). The enigma of Neoproterozoic giant ooids— Fingerprints of extreme climate?. Geophysical Research Letters, 47, e2019GL086146. https://doi.org/ 10.1029/2019GL086146
Koeshidayatullah A, Trower EJ, Xiaowei L, Mukerji T, Lehrmann DJ, Morsilli M, Al-Ramadan K, Payne JL., Quantitative evaluation of the roles of ocean chemistry and climate on ooid size across the Phanerozoic: Global versus local controls. Sedimentology 2022, 69, 2486–2506, 10.1111/sed.12998
Mauz B, Bode T, Mainz E, Blanchard H, Hilger W, Dikau R, Zöller L. The luminescence dating laboratory at the University of Bonn: Equipment and procedures. Ancient TL 2002, 20, 53-61.
Mauz, B, Nolan, P, Appleby, PG. Technical note: Quantifying uranium-series disequilibrium in natural samples for dosimetric dating – Part 1: gamma spectrometry. Geochronology 4, 213-225, https://doi.org/10.5194/gchron-4-213-2022
Murray A, Arnold LJ, Buylaert J-P, Guérin G, Qin J, Singhvi A, Smedley R, Thomsen KJ. Optically stimulated luminescence dating using quartz. Nature reviews, Methods Primers 2021, https://doi.org/10.1038/s43586-021-00068-5
Guérin G, Mercier N, Adamiec G. Dose-rate conversion factors: update. Ancient TL 2011, 29(1) 5-8.
Guérin G, Mercier N. Preliminary insight into dose deposition processes in sedimentary media on a scale of single grains: Monte Carlo modelling of the effect of water on the gamma dose rate. Radiation Measurements 2012, 10.1016/j.radmeas.2012.05.004.
