# Supplementary material for: Where did the river go? Testing the hypothesis of rivers discharging into the Gulf of Sirt (East Mediterranean) during the late Pleistocene
Source: PLoS One. 2025 Oct 8;20(10):e0331681. doi: 10.1371/journal.pone.0331681 (PMC12507219; doi:10.1371/journal.pone.0331681)
Supplement: Attachment — Submitted filename: Response to Reviewers.docx [file pone.0331681.s004.docx]

**Reviewer’s comment in black, our response in purple**

Reviewer#1

described methods (e.g., sample numbers, thin section analysis) are briefly mentioned and should be described in greater detail for reproducibility

We have followed standard procedures by examining 30 μm thick rock sections using a polarising microscope. Results including sample numbers are listed in Table S1. Site reference of samples is listed in Table S1 and shown in Fig S3.

Optical dating is mentioned, but the key details and uncertainties are only in the supplement. It would help to briefly summarize them in the main text.

In the study area all MIS 5/4 deposits are pure carbonate sediments. Only one sample contained quartz and could be used. Optical dating of quartz is a standard technique (Murray et al 2021). We feel that the description of a standard technique for one sample only does not need space in the main text. We have expanded the description in the Supporting Information.

The satellite and subsurface data are mentioned, it's not always clear how they integrate with field observations.

We appreciate this comment and intuitively agree with the referee, but where in the text is the integration missing? – an insolvable task for those who wrote the text. Line numbers would help.

line 284 "cortex growth could have been resumed" – this sentence could be clearer

It now says: “...cortex growth could have resumed each time when...” line 305

Some references appear to be repeated or inconsistently cited—please check for duplicates and ensure consistency only where necessary.

We have checked the in-text citations and the reference list and found that all references cited in the text are included in the reference list, and that all listed references appear to be cited in the manuscript. The numbering of the references also appears to be sequential and consistent.

Reviewer#2

Explicitly link absence of coastal fluvial deposits (e.g., no incisions/evaporites in Qunnayyin) to the rejection of Sirt rivers. Contrast with Chott’s positive evidence (Fig 7).

We appreciate this comment. The referee wishes to see the absence of evidence enhanced in the text in order to reject the presence of the Sirt river. Where in the text should this enhancement happen? In the original submission we say that (a) the Qunnayyin depression’s floor does not show fluvial deposits and its western margin does not show cliff incision (line 197), (b) no topographic break was found in the ridge (l 205), (c) alkaline seawater is not reconcilable with freshwater from the floodplain (l 262), (d) no floodout deposits were found in the sabkha Kabirah (l 277); we say at the end: “T*here is no definite piece of evidence for or against a river, but the sum of our observations speak for a Saharan transport-limited ephemeral river discharging its water into the Qunnayyin depression* (l 287). Regarding contrast to Chott we say “*The Chott basins and adjacent coastal zone show features expected to arise from freshwater ponding in the depression and fluvial discharge on the coast*” (l 316). We are unsure how to improve the text.

Emphasize how the Irharhar-Chott pathway reconciles deep-sea geochemical data (e.g., core CP10) and LOVECLIM rainfall models

In l 315 (original submission) we say which data obtained from core CP10 point to the Chott region and cite Wu et al 2016 for data and interpretation. The LOVECLIM rainfall model results mentioned in the text are from Blanchet et al 2021. This study models rainfall for Tibesti and coastal Libya, not for the Chott. Regarding rainfall in the Chott we provided a qualitative explanation in l 326ff.

Ensure all figures cited (e.g., Fig 6).

Fig 6 is cited in line 313.
Define key terms (e.g., "transport-limited ephemeral river").

Transport limitation is typical for ephemeral rivers as they tend to have insufficient water available to transport the sediment available for transport. This is described in l 313. We are unsure about other key terms to define.
Fix minor errors (e.g., duplicate "the" in line 260).

Thank you – done.

Briefly note how aquifer salinity affects "green corridor" viability near Sirt.

Our study did not focus on evidence or absence of a green corridor. The idea circulates in literature and had to be mentioned by us in the introduction. In the conclusion we point out that the aquifer in Sirt’s coastal plain is brackish, hence unsuitable to sustain human settlements in pre-Holocene times.
